# The Interplay between PolyQ and Protein Context Delays Aggregation by Forming a Reservoir of Protofibrils

**DOI:** 10.1371/journal.pone.0000111

**Published:** 2006-12-27

**Authors:** Donatella Bulone, Laura Masino, David J. Thomas, Pier Luigi San Biagio, Annalisa Pastore

**Affiliations:** 1 National Research Council (CNR), Istituto di Biofisica di Palermo, Palermo, Italy; 2 National Institute for Medical Research, London, United Kingdom; 3 Scientific Software Solutions, Paisley, United Kingdom; Fred Hutchinson Cancer Research Center, United States of America

## Abstract

Polyglutamine (polyQ) diseases are inherited neurodegenerative disorders caused by the expansion of CAG codon repeats, which code for polyQ in the corresponding gene products. These diseases are associated with the presence of amyloid-like protein aggregates, induced by polyQ expansion. It has been suggested that the soluble aggregates rather than the mature fibrillar aggregates are the toxic species, and that the aggregation properties of polyQ can be strongly modulated by the surrounding protein context. To assess the importance of the protein carrier in polyQ aggregation, we have studied the misfolding pathway and the kinetics of aggregation of polyQ of lengths above (Q41) and below (Q22) the pathological threshold fused to the well-characterized protein carrier glutathione S-transferase (GST). This protein, chosen as a model system, is per se able to misfold and aggregate irreversibly, thus mimicking the behaviour of domains of naturally occurring polyQ proteins. We prove that, while it is generally accepted that the aggregation kinetics of polyQ depend on its length and are faster for longer polyQ tracts, the presence of GST alters the polyQ aggregation pathway and reverses this trend. Aggregation occurs through formation of a reservoir of soluble intermediates whose populations and kinetic stabilities increase with polyQ length. Our results provide a new model that explains the toxicity of expanded polyQ proteins, in which the interplay between polyQ regions and other aggregation-prone domains plays a key role in determining the aggregation pathway.

## Introduction

Polyglutamine (polyQ) diseases are caused by the expansion of CAG codon repeats resulting in extended polyQ tracts in the expressed proteins [1]. This family of inherited neurodegenerative disorders includes Huntington's chorea, spinobulbar muscular atrophy, dentatorubral-pallidoluysian atrophy, and spinocerebellar ataxias (SCAs) 1, 2, 3, 6, 7, and 17. The polyQ region is the only common feature of the proteins associated to these diseases, that are otherwise totally unrelated [2]. In affected individuals, the polyQ tract is expanded above a threshold of ca. 35 consecutive glutamines, resulting in the aggregation of the mutant protein and the consequent formation of intranuclear inclusions [3].

Although the role of aggregation and fibre formation of expanded polyQ proteins has not yet been established clearly, protein misfolding and aggregation are accepted to be central issues for understanding the molecular mechanisms of these pathologies [4]. *In vitro* studies have shown that polyQ aggregation depends on protein concentration, repeat-length, and time and that it occurs with a nucleation-dependent mechanism [5]–[7]. A conformational transition from random coil to β-sheet, which share most of the features typical of amyloids, takes place during the process of fibre formation [8]–[10]. However, detailed structural information on polyQ aggregates is still unavailable and the steps leading to the assembly of mature fibres are not yet fully understood.

Kinetic studies of polyQ protein aggregation *in vitro* have shown that formation of amyloid or amyloid-like fibres generally occurs via fibrous intermediates that can have distinct morphologies [10]–[13]. Cell biology studies have suggested that these early aggregates or proto-fibres rather than the insoluble aggregates are the main cytotoxic species, with mature fibres having a beneficial role for neuronal cells [14]–[17]. This hypothesis has been formulated also for other neurodegenerative diseases related to protein misfolding and aggregation, such as Parkinson's and Alzheimer's diseases and the transmissible spongiform encephalopathies. Increasing evidence suggests that soluble aggregates-mediated toxicity might be a common pathogenesis mechanism for these disorders [18]–[23]. The characterisation of the early phases of fibrillation is therefore critical for understanding the molecular causes of pathogenesis.

Another central issue is the relationship between the polyQ tracts and other regions of the proteins that host them. Although polyQ expansion is certainly the main factor responsible for protein aggregation, various studies have demonstrated that the protein context plays an important role in determining the stability and solubility of polyQ peptides and may modify and contribute to the aggregation process [24]–[27]. It is therefore important to investigate the properties of polyQ when flanked by different protein sequences in order to mimic their effect on aggregation.

In this work, we have studied the aggregation properties of a model system consisting of two polyQ peptides of different lengths, one below (22 glutamines) and one above (41 glutamines) the pathological threshold, fused to glutathione S-transferase (GST). GST was chosen because it is a well characterised protein of known structure, which can be used to mimic the environment surrounding the polyQ region in full-length proteins, thus providing an ideal model to assess the effects of protein context. GST-polyQ proteins have been instrumental in establishing that, prior to aggregation, polyQ is disordered regardless of its length, and in assessing the hypothesis of a structural threshold between short and long polyQ sequences [25], [28].

Here, we have investigated the aggregation pathway of these fusion proteins and compared their polymerization kinetics with those of GST. Using complementary biophysical techniques, such as optical spectroscopy methods and both dynamic and static light scattering, we have been able to characterize the structural properties of the proteins during the process of aggregation, to estimate the timescale of aggregation, to assess the presence of different species and measure their size and relative populations. Our results show that the presence of a carrier may change significantly the aggregation pathway of polyQ and that proto-fibril formation may compete with bigger insoluble aggregates.

## Results

### The presence of the polyQ tail influences the aggregate population

**Figure 1:**
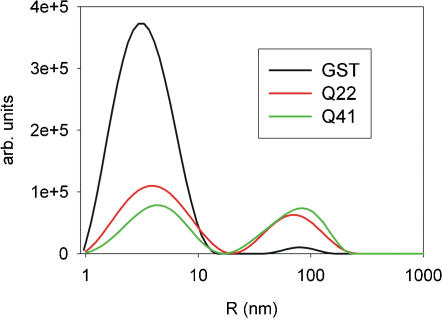
Comparison of the intensity-weighted size distribution of GST (black line), GST-Q22 (red line) and GST-Q41 (green line) at 90° scattering angle. The measurements were carried out on 20 µM protein samples and at 20°C.

The aggregation state of GST, GST-Q22 and GST-Q41 was first characterized by dynamic light scattering (DLS) to obtain information about the size of the species present in solution. Analysis of the correlation function shows a bimodal distribution, for all three samples which therefore contain two distinct populations (Figure 1). The hydrodynamic radii of the dominant species are 4–7 nm. GST is well known to form stable dimers in the whole range of concentrations considered in this study, having a hydrodynamic radius of 3.7 nm (gyration radius of 2.9 nm) as calculated with the assumption of a globular shape of the molecule. The observed experimental radius measured for GST (4.2±0.5 nm) is therefore in excellent agreement with the presence of the dimer as the minimal unit. Marginally larger radii were observed for GST-Q22 and GST-Q41 (5.4±0.5 and 6.3±0.5 nm). These values can be explained by the progressive failure of the assumption of an isotropic globular shape, which is expected for the two samples in which the polyQ tail is unstructured [25].

The second population corresponds to species with much larger molecular weights. Their hydrodynamic radius in the GST sample is 80±10 nm which corresponds to a soluble aggregate of ca. 3500 dimeric units, but their population is so small as to be practically negligible. We estimated the numeric ratio between the soluble aggregate and the dimer to be ca. 1∶7000000, which accounts for ca. 0.1% of the total population by mass. In GST-Q22 and GST-Q41, the soluble aggregates have averaged radii of 91±4 and 96±4 nm respectively, which correspond to ca. 5000–7000 dimeric units. The aggregate populations are also small in these samples. This explains why these species were not detected by NMR or CD in previous studies [25] but can be observed by a technique as sensitive to aggregation as DLS: large aggregates scatter light much more than small species and therefore even small populations of them will be detectable (with the same argument, we cannot exclude the presence of minute populations of species with radii close to that of the dimer). Despite the minor populations of the soluble aggregates, a clear trend was observed in all samples studied (from three different protein batches): the numerical ratios between the larger and the smaller species are of about 1 to 140000±35000 and of 1 to 32000±8000, which correspond to ca. 3% and 11% of the total populations by mass of GST-Q22 and GST-Q41, respectively, suggesting that the relative populations of the soluble aggregates correlate with the length of the polyQ tract. No significant time dependence of these ratios was observed over a period of several months and the range of concentrations considered (6–20 µM).

These results strongly indicate an intrinsic tendency of polyQ to promote aggregation. While not affecting the size, which seems to be determined by the carrier, the length of the polyQ tract influences the relative populations of the aggregates.

### The presence of a reducing agent discriminates between different types of aggregation

**Figure 2:**
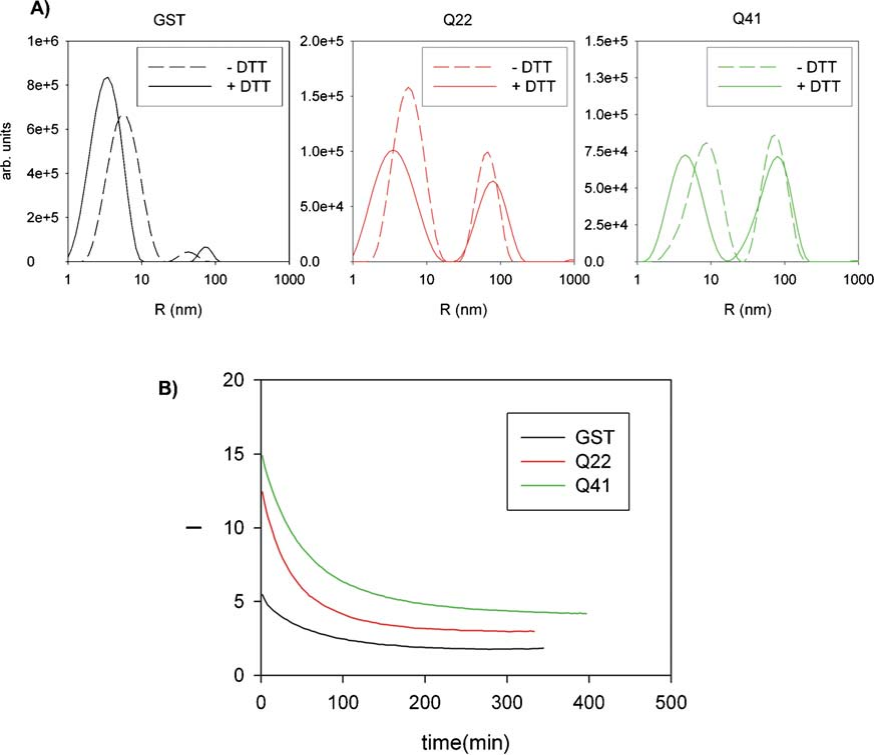
Effect of an antioxidant on aggregation. **A)** Comparison between the intensity-weighted size distributions obtained in the absence and in the presence of DTT. **B)** Time course of light scattered intensity at 90° angle upon addition of DTT to fresh protein samples. GST, GST-Q22 and GST-Q41 are shown using black, red and green lines, respectively. The measurements shown were carried out at 20°C using 20 µM samples.

We then explored the nature of the aggregation. GST has four cysteines that are not involved in intramolecular sulphur bridges and can thus bond intermolecularly and promote covalent aggregation [29]. This phenomenon would be unwanted in the present study since it would depend on the specific choice of the protein carrier and not on the polyQ tract. To make sure that the presence of DTT effectively inhibits the formation of disulphide bridges, and to estimate the extent of their interference with the formation of non-covalent polyQ aggregates, we compared measurements in the presence and in the absence of a reducing agent (Figure 2A). In experiments carried out in the absence of DTT, the correlation function of GST showed a distribution which corresponds to a species with a weighted average radius of 7.5 nm, which corresponds roughly to a dimer of the dimeric form, and only minor traces of a species of 50 nm. This is much smaller than the one observed in the presence of DTT. The size distribution of GST-Q22 and GST-Q41 is bimodal with average radii of 7.2 and 74 nm, and 10 and 86 nm, respectively. We did not observe a dependence of their relative populations on the protein concentration, at least in the range of concentrations explored (5–40 µM). When an excess of freshly prepared DTT (1 mM) was added to the samples without DTT, a progressive decrease of scattered light was seen and followed until a steady state was reached (Figure 2B), indicating a decrease of the average dimensions of the sample species in response to the anti-oxidant effect of DTT. The final state is indistinguishable from that observed in samples always treated with DTT.

These results indicate that covalent aggregation of GST can compete with the non-covalent aggregation observed under reducing conditions producing different, smaller soluble aggregates. The effect can, however, be effectively reversed by addition of DTT. All the following studies were carried out in the presence of DTT.

### Thermally-induced aggregation occurs at higher temperatures in GST-polyQ proteins

**Figure 3:**
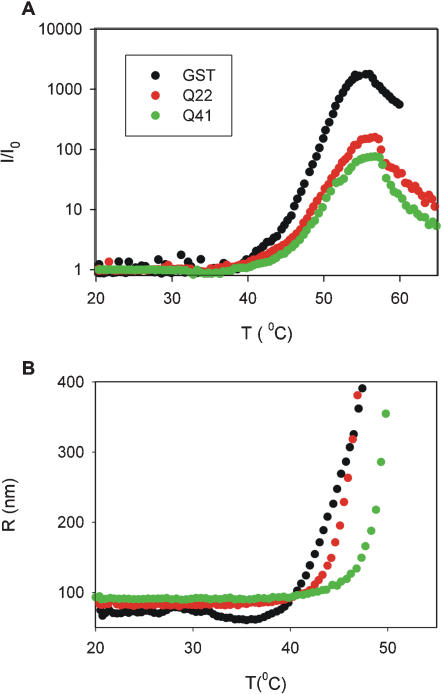
Effect of temperature on aggregation. **A)** Light scattered intensity at 90° angle registered during the temperature scan. Data relative to different proteins are normalized by dividing for the respective initial values of scattered intensity. **B)** Weight-average radius of the largest species, as obtained by CONTIN analysis [38], followed during the temperature scan.

To study further the nature and the mechanism of aggregation, the thermal stabilities of GST, GST-Q22 and GST-Q41 were assessed by DLS, recording thermal unfolding profiles. Heat should promote aggregation if this is mainly hydrophobic. Thermal denaturation of GST causes a temperature-dependent increase of scattered light intensity, concomitant with a size increase of the aggregates (Figures 3A and B). The temperature at which deviation of the signal from the baseline starts appearing depends on the sample concentration. For GST, it is in the range of 38–48°C for 6–20 µM protein concentrations (data not shown). The increase continues up to ca. 55–58°C. Above this temperature, the signals decrease, concurring with the appearance of a visible precipitate at the bottom of the cuvette. This behaviour reflects the opposing trends of an increase of scattered light due to the increasing dimensions of the species present in solution and their loss from the same solution by precipitation. The event is irreversible, as previously described for GST [29].

DLS temperature scans measured for GST-Q22 and GST-Q41 show similar features (Figure 3). Thermal unfolding is irreversible also for these samples, and the starting temperature of aggregation has an appreciable dependence on concentration (data not shown). However, a clear difference is observed between the three samples: at the same protein concentration, aggregation initiates at higher temperature for the polyQ fusion proteins than for GST. Interestingly, GST-Q41 starts aggregating at a higher temperature than does GST-Q22. The extent of these differences depends on the history of the samples and on the scanning rates, but the qualitative behaviour is consistent and reproducible.

Our results confirm that thermally induced destabilization of GST causes irreversible aggregation, and indicate that the same is true for GST-polyQ proteins. The temperature of this transition is influenced both by the presence and by the length of the polyQ tract. This suggests that, although undergoing the same process, the kinetics of aggregation of polyQ fusion proteins are slower than those of GST.

### GST-polyQ fusion proteins form a reservoir of soluble aggregates

**Figure 4:**
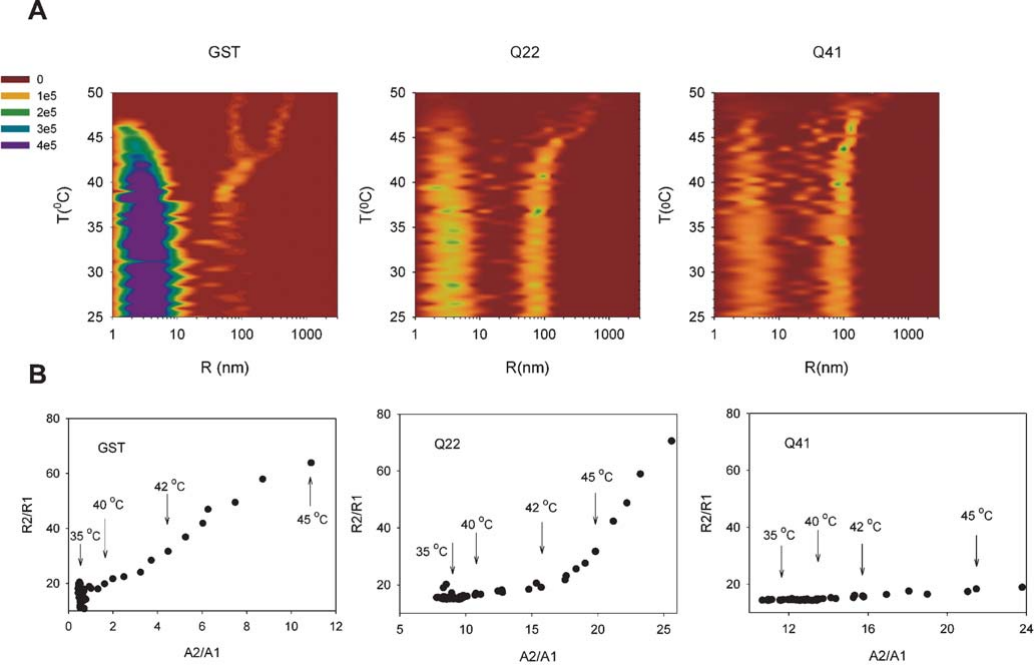
Analysis of the species size during the temperature scan. **A)** Contour plots of intensity-weighted size distribution during the temperature scan. **B)** Size ratio of large to small species, R_2_/R_1_, plotted versus the ratio of their relative light scattering contribution, A_2_/A_1_ during the temperature scan. The corresponding temperatures are indicated. **C)** Weight-averaged radius of the largest species, as obtained by CONTIN analysis [38], in the course of temperature scan.

To follow in more details the early steps which precede the final catastrophic events after which precipitation starts to occur, the behaviour of the individual species in solution was monitored by a three dimensional representation of the intensity-weighted size distribution during the temperature scan monitored by DLS (Figure 4A). The plot clearly shows the simultaneous presence of the minimal dimeric form and of the soluble aggregates. The dominant and almost exclusive species present in the GST sample remains the minimal dimeric unit (of ca. 4 nm radius) up to 38°C, with only a minor contribution of a larger species. The populations of the soluble aggregates of GST-Q22 and GST-Q41 are more appreciable at all temperatures. For all three samples, there is no significant appearance of species of dimensions intermediate between the dimer and the soluble aggregates of 80–96 nm radii. At high temperature, the size of the large species increases exponentially (note that the x axes in Figure 4A are logarithmic), suggesting that there is a progressive and direct scavenging of the dimer by the soluble aggregates. Eventually, the signal from the large species disappears completely as they grow into an insoluble large aggregate and fall out of solution. The temperatures at which the size starts to increase and at which the signal disappears are different for the three samples and are progressively higher as a function of the polyQ length. It is also clear from the plots that the size growth of the soluble aggregate at the expense of the dimer observed at high temperatures is larger for GST than for GST-Q22 and GST-Q41, which increase more slowly.

A two-dimensional plot of the ratios of the average dimensions of the two main species present in solution (R_2_/R_1_, where species 1 refers to the minimal dimeric species, of ca. 4–7 nm radius, and species 2 is the soluble aggregate, originally of ca. 80–90 nm radius) *vs.* the ratio of the respective contributions to the correlation function amplitude (A_2_/A_1_) provides a complementary description (Figure 4B). For GST, there is a close to linear increase of the dimensions of the aggregates at increasing A_2_/A_1_ ratios in the whole range of temperatures up to signal disappearance. For GST-Q22 and GST-Q41 instead, the amplitude increment is not paralleled by an increase of the radii ratios, which remain almost constant in the range 35°C–42°C, although the overall scattering intensities increase exponentially over the whole range of temperature (cf. Figures 3A and 4B). This strongly suggests that GST aggregation proceeds through intermediate formation of soluble species which grow immediately into large insoluble aggregates. Their population does not however increase and the large aggregates grow so much that they start to precipitate. On the contrary, over the same temperature range, GST-Q22 and GST-Q41 convert first the minimal dimeric unit into the soluble aggregate, whose population rather than size increases. Only when this intermediate of aggregation is significantly populated does the process of aggregation proceed to formation of large aggregates.

These results indicate that aggregation occurs via an intermediate which behaves as a seed (or nucleus) for further aggregation. However, the mechanism of aggregation of GST is different from that of GST-Q22 and GST-Q41, which form a reservoir of soluble aggregates before proceeding to the formation of insoluble large species.

### Temperature induced aggregation is associated with a conformational transition

**Figure 5:**
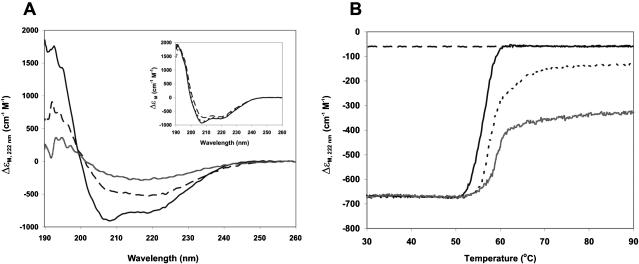
The effect of temperature on the far-UV CD spectra. **A)** Far-UV CD spectra of GST-Q41 recorded at different temperatures: 20°C (continuous black line), sample incubated at 51°C for 30 minutes (dashed line), and then incubated at 68°C for 1 hour and 20 minutes (grey line). Protein concentration was 4 µM. **Inset**: Far-UV CD spectra of GST (dashed line), GST-Q22 (dotted line), and GST-Q41 (continuous line) recorded at 20°C. **B)** Far-UV CD thermal scans of GST (continuous black line), GST-Q22 (dotted line), and GST-Q41 (grey line) recorded at 222 nm with a heating rate of 1°C/min. The curves were normalized to the intensity of the GST sample at 20°C for comparison purposes. The cooling profile of GST (dashed line) was recorded with the same rate and is added as an example of irreversibility. A similar behaviour was observed for the other two proteins. Protein concentration was 20 µM in all samples.

The process was monitored independently using far-UV CD to follow the conformational state of the samples during thermally induced aggregation. The CD spectrum of GST at 20°C is typical of an α/β protein, with a predominance of the α-helical signal, and in agreement with literature data (Figure 5A) [29]. At 20°C, the spectra of GST-Q22 and GST-Q41 are very similar to that of GST, but have a slightly higher content of random coil conformation due to the presence of the polyQ tail, which has been proven to be highly flexible and not to interact with the carrier protein (Figure 5A, inset) [25]. For all three proteins, spectra measured at higher temperatures showed that heating induces a secondary structure transition from an α/β structure to a conformation with a higher β-sheet content. Such a transition has been described for several proteins that are known to form fibres and are associated with misfolding diseases [30]. At the same time, the overall signal decreases irreversibly. Visual inspection and electron microscopy images of the samples after the scan (see below) show that this irreversible process is aggregation, in agreement with LS measurements. The CD signal decrease therefore arises from two interconnected phenomena: a secondary structure variation and protein aggregation, which eventually causes precipitation and loss of the signal.

When thermal scans monitored at a fixed wavelength (222 nm) were registered, we observed the same phenomenon as in DLS measurements: there is a clear difference in the transition midpoints of the three samples, with GST having a transition at lower temperature (Figure 5B). The amplitude of the apparent transition is smaller for GST-Q41 than for GST-Q22 and, even more so, for GST. A final signal intensity similar to that of GST is reached also by GST-Q22 and GST-Q41, but only after incubation at high temperature for several hours (data not shown).

Quantitatively, the temperature at which formation of the insoluble aggregates starts being observable by DLS appears lower than that at which we observe a conformational transition by CD. This difference is partly a consequence of the slower scanning rate imposed by DLS measurements, which lead to work at quasi-equilibrium conditions. However, even taking this effect into account, we observed consistently that the structural transition occurs at a temperature at which the dimer ceases to influence the DLS measurements. This delay suggests that the two techniques record different albeit interconnected events. The β-rich signal does not arise from the dimer but becomes detectable only when the aggregates become the dominant species in solution. We cannot, on the other hand, infer anything on the structure of the aggregates while they are in co-presence with the dimers, since their populations are too small to influence the CD signal.

These observations provide direct information about the structural changes occurring upon aggregation and confirm the presence of different kinetics of aggregation due to the presence of the polyQ tail.

### Studying further the kinetics of aggregation

**Figure 6:**
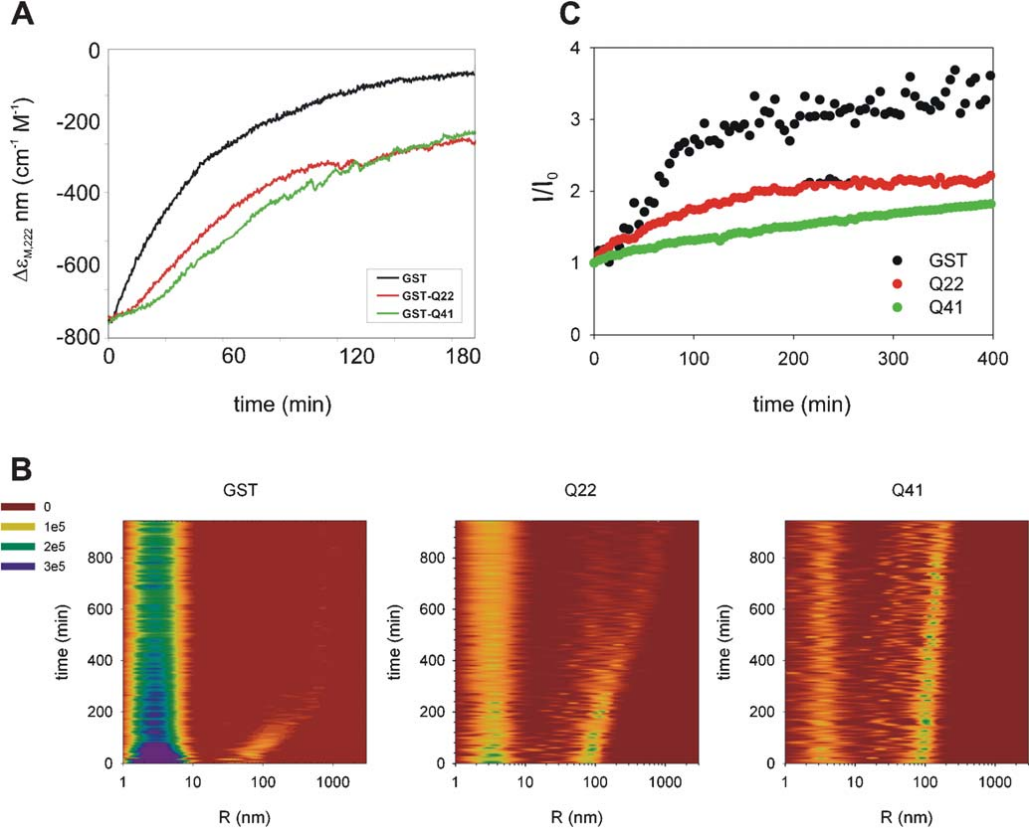
Kinetics of aggregation. **A)** Far-UV CD time scans of GST (black line), GST-Q22 (red line), and GST-Q41 (green line) recorded at 50°C. The signal changes, monitored at 222 nm, arise from both protein aggregation and the associated conformational transition. The curves were normalized to the intensity of GST for comparison purposes. Protein concentration was 4 µM in all samples. **B)** Contour plots of intensity-weighted size distribution of samples quenched at 37°C. **C)** Time course of light scattered intensity at 90° angle of samples quenched at 37°C.

To quantify the differences in behaviour of GST, GST-Q22, and GST-Q41, time scans were recorded at fixed temperatures using both LS and CD. The far-UV CD data, recorded at temperatures close to the beginning of the thermally induced transitions, showed that the predominantly helical signal converts, as a function of time, into a β-rich spectrum, as observed during temperature scans (data not shown). The signal intensity also decreases with time, in concomitance with the formation of an insoluble precipitate (Figure 6A). The kinetics of the α to β transition and aggregation are temperature dependent and are slower at lower temperature, as expected for a hydrophobic process. Conformational changes and aggregation occur with faster kinetics for GST, followed by GST-Q22 and then GST-Q41. The kinetics are characterised by a lag phase, followed by an exponential decay phase, typical of nucleation-dependent polymerization [31]. At 50°C, the lag phase measured for GST is approximately 3 min, whereas GST-Q22 and GST-Q41 have lag phases of approximately 19 and 26 min, respectively. The estimated average values of the apparent transition half lives at 50°C are 31, 40, and 67 (+/−3) min for 4 µM samples of GST, GST-Q22, and GST-Q41, respectively (Table 1).

**Table 1:**
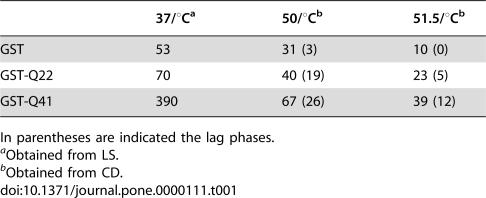
Half lives (min) of GST, GST-Q22 and GST-Q41 as estimated from LS and CD data recorded at different temperature.

	37/°C^a^	50/°C^b^	51.5/°C^b^
GST	53	31 (3)	10 (0)
GST-Q22	70	40 (19)	23 (5)
GST-Q41	390	67 (26)	39 (12)

In parentheses are indicated the lag phases.

a Obtained from LS.

b Obtained from CD.

When the aggregation kinetics were followed by DLS, the scattered light intensity was recorded at 37°C, which is the temperature at which the exponential increase of scattered light starts. This temperature is significantly lower than the one used to detect conformational changes by CD, but we wanted to make sure we could follow with each technique the early stages of aggregation. A longer persistence of the soluble aggregates of GST-Q41 is very clear in a three-dimensional representation (Figure 6B). Intensity plots measured at different time intervals show that GST aggregates faster, followed by GST-Q22, and finally GST-Q41, with half lives of 53 min, 70 min and 390 min (Figure 6C). These values are in excellent qualitative agreement with those obtained by CD, which, having been recorded at higher temperatures, are smaller. The conversion of the GST soluble aggregates into larger species is also much faster, over the same time interval, than that of the polyQ fusion proteins. Measurements performed with sample concentrations in the range 6–30 µM indicate a clear dependence of aggregation kinetics on concentration (data not shown).

Taken together, the CD and LS data strongly suggest that, although the presence of the polyQ tail does not affect the secondary structure of GST, it has a striking effect on the time scales of formation of insoluble aggregates. The polyQ tracts seem to delay the aggregation process of GST in a polyQ length-dependent manner.

### The tendency to form fibrils increases with the polyQ length

**Figure 7:**
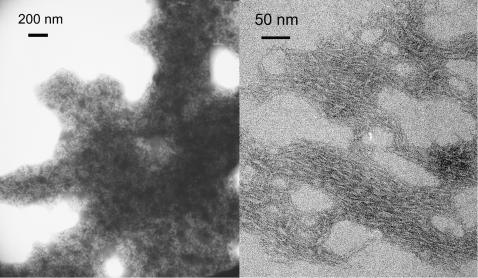
Visualization of the large insoluble aggregates as observed by EM. GST (left) and GST-Q41 (right) samples were analysed immediately after the kinetics at 50°C.

A different behaviour of the three samples could also be deduced from direct visual inspection of the samples. Immediately after the kinetic studies, only GST was cloudy, whereas the other two samples showed physical precipitation at the bottom of the cell or became opalescent only after several hours at high temperature (data not shown). Inspection of the samples by EM showed the presence of fibrillar aggregates only for GST-Q41 (Figure 7). They were typically 10–50 nm long and narrower than 5 nm. GST-Q22 and GST showed amorphous aggregates that were usually too big even to be visualised in the EM grids. While we cannot exclude that also these samples could form fibres if the correct conditions were found, we must conclude that longer polyQ tails facilitate fibre formation in GST fusion proteins, in agreement with what has been observed for isolated polyQ peptides [28].

## Discussion

An increasing number of diseases that result in neuronal death have been associated with protein misfolding and aggregate formation. Accumulating evidence strongly supports the view that cytotoxicity arises from the presence of soluble aggregates and/or prefibrillar species, rather than from the fibrillar species which form the insoluble aggregates accumulated in the cell either as amyloid fibres or as amorphous deposits. In this newer model, the insoluble species would serve only as an escape route for smaller aggregates. It is therefore essential to characterize the species involved and the misfolding pathways that relate them in order to understand the mechanism of aggregation.

Here, we have adopted a model system which makes use of GST as a carrier to investigate the role of protein context in the aggregation pathway of polyQ. We have shown that the presence of polyQ tails results in the trapping and stabilization of a small but well defined population of soluble aggregates. Interestingly, the size of these species is defined by the carrier protein and not by the polyQ tract, as judged from their presence already in the GST sample. They must reflect an intrinsic tendency of this protein to misfold during over-expression and/or purification, a behaviour possible also in proteins as soluble as GST, which is known to undergo irreversible transitions upon stress conditions [29]. When the polyQ tail is added, the population of aggregation intermediates increases by 1–2 orders of magnitude (depending on the polyQ length) already at room temperature, without the appearance of additional species. Although not necessarily structurally and morphologically similar, the soluble aggregates observed in the three samples seem to have similar hydrodynamic radii. This suggests that the polyQ tract increases the probability of misfolding but the carrier protein acts as the template for aggregation formation. We do not observe significant dimer-to-aggregate inter-conversion at room temperature, in agreement with the observation that no aggregation was detected by CD and NMR for the same constructs over a period of months [25].

The pathway of thermal aggregation of the three samples was followed by CD, LS and both optical and electron microscopy. These techniques provided complementary information. LS, being highly sensitive even to minute populations of aggregates whose capacity to scatter light increases with molecular size [32], helped us to characterize the size of the species present in solution. CD provided a description of the secondary structure of the populations dominant in solution, whereas optical and electron microscopies gave us information on the morphology of the insoluble aggregates. The model which comes out from LS data is that aggregation proceeds through a two-step irreversible conversion of the three species, the minimal dimeric units, a soluble high molecular weight species and large insoluble aggregates of dimensions too large to be detected even by LS (i.e. roughly≥10 µm). Before disappearing into the large insoluble aggregates, an appreciable population of dimer is trapped in the intermediate state which becomes increasingly more populated. The soluble aggregates are thus an integral part of the aggregation pathway: they appear to behave like ‘sticky glue balls’ which scavenge dimers remaining in solution, and thus act as the foci for further aggregation. The process does not consist of a simple unfolding event but, as observed by CD, is a complex transition in which aggregation is tightly associated with an irreversible structural transition which results in a significant enrichment of β content.

The effect is, however, not the same for all the samples tested, as already reported before [25]. Irreversible aggregation of GST occurs either at lower temperatures or, when following the process at constant temperature, with faster aggregation kinetics than those of GST-Q22 and GST-Q41. This suggests that the two pathways dimer-to-soluble aggregate and from the soluble-to-insoluble aggregate have a similar efficiency only for GST, whereas one of them is disfavoured in GST-Q22 and GST-Q41 with consequent, if transient, formation of a reservoir of soluble high molecular weight intermediates or protofibrils. These results could look somewhat counterintuitive and in direct conflict with what is observed when the polyQ tail is proteolytically cleaved from the carrier: the cleaved polyQ peptides have aggregation kinetics which depend on the polyQ length so that, even at room temperature, Q41 aggregates must faster than Q22 [28]. A similar behaviour has been observed for polyQ tracts fused to CRABP I, a highly soluble protein with a reversible unfolding pathway [33]. The main difference between these examples and our GST model system is that, under destabilizing conditions, unmodified GST is able to undergo irreversible aggregation on its own. This strongly suggests that, when together, the two distinct elements GST and polyQ, each with intrinsic tendencies to aggregation, do not behave independently but mutually affect each other's behaviour.

The importance of protein context in modulating the behaviour of polyQ is largely supported by independent evidence [24], [26], [27], [33], [34]. The effect seems to work both ways. Soluble carriers are known to solubilize the insoluble polyQ and to make it stable in solution for several months. Well known examples are myoglobin, CRABP I and GST itself. Addition of a proline-rich extension to a polyQ tract has also been shown to decrease its tendency to aggregate [35]. Conversely, protein domains outside the polyQ tract have been shown to increase the tendency of polyQ to misfolding: cellular studies of the aggregation propensity of expanded and non-expanded ataxin-1 and ataxin-3 have shown that, while promoted by polyQ expansion, aggregation can be noticeably reduced by deletion of such domains or their replacement with sequences with no known tendency to aggregate [34], [36]. Incorporation of a polyQ tract into a loop of the stably folded chymotrypsin inhibitor 2 (CI2) has also been shown to lead to formation of misfolded dimeric and trimeric species [37]. It is therefore not entirely surprising that, in addition to increasing the probability of misfolding and causing structural destabilisation, polyQ can alter the kinetics of aggregation of its carrier proteins, thus having an effect on the time scale of aggregation.

The effect can be due to different causes. The flexible polyQ tail, which, before aggregation, fluctuates freely in solution, could mask the surface which promotes GST aggregation, decreasing the probability of effective collisions of this region with other molecules. This would disfavour further transition to the insoluble aggregates, thus slowing the kinetics. The polyQ tail could also increase the stability of the GST aggregation intermediate by transiently interacting with the carrier. It might be more difficult to promote transition of both the polyQ tail and the globular GST to the β-rich structure of the large aggregate for steric hindrance reasons. Recent theoretical studies also indicate that merely adding a large mass to the terminus of a protein tends to stabilise the fold (DJT, unpublished data).

The presence of larger reservoirs of soluble aggregates or protofibrils along the aggregation pathway observed for the polyQ fusion proteins could be correlated with their increasing tendency to form ordered fibres, a feature that is easily observed for polyQ peptides but not for GST. The trapped intermediates, which kinetically prevent the immediate appearance of large insoluble aggregates, could lead more easily to well-ordered fibrillar structures, as for crystal formation, where slower growth conditions are generally beneficial once nucleation has occurred.

How can our observations be related to ‘real’ polyQ proteins? The results presented here are coherent both internally and with what is known about the behaviour of polyQ proteins. GST, which was originally chosen mainly because it is a well characterised globular protein for which both the structure and the unfolding properties are known, turned out *a posteriori* to be an ideal model for naturally occurring polyQ proteins. Its ability to aggregate irreversibly through formation of a β rich conformation mimics the behaviour of at least two of the nine known proteins linked to polyQ diseases. Both ataxin-1 and ataxin-3 contain globular motifs, the AXH and the Josephin domains, which have strong intrinsic tendencies to form non-covalent aggregates [27], [34]. It is tempting to suggest that the tendency of protein context to stabilize larger populations of soluble aggregates or proto-fibrils is an important and more general behaviour, which could be at the basis of polyQ pathologies. If, as now widely believed, the toxic species is the soluble aggregates and/or the protofibrils rather than the insoluble aggregates, this model could help to explain why longer polyQ tracts are more toxic than short ones. More work will be needed to extend this model to more specific examples.

## Materials and Methods

### Samples preparation

The DNA sequences coding for 22 and 41 glutamines were cloned into a pGEX-4T1 plasmid vector containing *Schistosoma japonicum* GST with a 21-residue linker at the C-terminus, as described before [25]. The GST-fusion proteins were expressed in *E. coli* strain BL21 and purified by affinity chromatography using glutathione-agarose beads (Amersham Pharmacia). The purity of the samples was assessed by SDS-PAGE and mass spectrometry. Protein concentrations were determined using UV absorption, with calculated extinction coefficient at 280 nm of 40920. The buffer used was 40 mM sodium phosphate, pH 6.5, and 1 mM dithiothreitol (DTT) to prevent cysteine oxidation. The experiments performed under non-reducing conditions were performed in the same buffer, in the absence of DTT.

### Static and dynamic light scattering

Before each measurement, the samples were filtered using 0.2 µm pore diameter membranes (Sartorius), put into dust-free optical cells and placed into a thermostated cell compartment of a Brookhaven Instruments BI200-SM goniometer to carry out the measurements. The temperature was controlled within 0.1°C using a thermostated recirculating bath. The light scattered intensity and time autocorrelation function were measured by using a Brookhaven BI-9000 correlator and a 100 mW Argon laser (Melles Griot) tuned at λ = 514.5 nm. The spatial resolution is defined by the scattering vector q = 4πnλ_0_^−1^sin(θ/2), where *n* is the refraction index of the solution, λ_0_ is the wavelength of the incident light, and θ is the scattering angle. Static light scattering data were corrected for the background scattering of the solvent and normalized by using toluene as calibration liquid. In DLS experiments, the correlator was operated in the multi-channel mode. To assess reproducibility, each experiment was repeated at least three times using independent batches of proteins. The size and relative populations indicated throughout the manuscript are averaged over the measurements.

### Data analysis

The field autocorrelation function, g^(1)^(τ), was obtained by measuring the intensity correlation function and analyzed by using CONTIN [38], in order to determine the distribution of relaxation times according to:
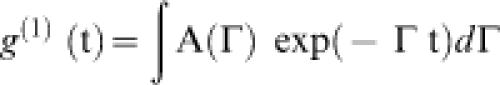
where **A**(***Γ***) denotes the contribution amplitude of the mode with characteristic time ***Γ^−1^***. The latter is related to the diffusion coefficient by:
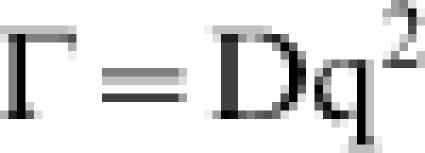



The hydrodynamic radius is obtained by the Stoke-Einstein relationship D = k_B_T/6πηR_H_. The simpler cumulative analysis, which gives the average value and the width of the size distribution [39], can be strictly applied only for GST samples under non-aggregating conditions, where large species are negligible and a modal distribution is observed. For GST-Q22 and GST-Q41 or even for GST samples under aggregating conditions, a contribution of two different particle populations was observed always.

### Circular dichroism

CD measurements were performed on a Jasco J-715 spectropolarimeter equipped with a PTC-348 Peltier temperature control system, which allows a maximal error of 0.1°C. CD spectra were recorded using quartz cuvettes (Hellma) with pathlengths of 1 mm. Protein samples were in 40 mM phosphate pH 6.5, 1 mM DTT, with protein concentrations of 4–20 µM. CD intensities are presented as the CD absorption coefficient calculated using the molar concentration of the proteins (*Δ*ε_M_). Thermal scans were measured by increasing temperature from 20 to 90°C at 1°C/min or at 10°C/hour. Reversibility was assessed by cooling to 20°C using the same rate. Temperature and time scans were recorded by monitoring the CD signal at 222 nm.

### Electron microscopy

The samples used for CD temperature or time scans were directly analysed by EM, applied to carbon coated grids and stained with 1% sodium silico-tungstate (pH 7). The grids were viewed under minimal dose, accurate defocus conditions with a Jeol 1200EX operated at 100 kV.
